# Lower Extremity Trauma: A Multidimensional Reconstructive Approach with Hyperbaric Oxygen Therapy

**DOI:** 10.3390/jcm13051407

**Published:** 2024-02-29

**Authors:** Caterina Marra, Paola Pentangelo, Luigi Losco, Alessandra Ceccaroni, Alfonso Barbato, Carmine Alfano

**Affiliations:** 1 Plastic Surgery Unit, Department of Medicine, Surgery and Dentistry, University of Salerno, 84081 Baronissi, SA, Italy; camarra@unisa.it (C.M.); ppentangelo@unisa.it (P.P.);; 2 Plastic Surgery Unit, Azienda Ospedaliera Universitaria OO.RR. San Giovanni di Dio e Ruggi d’Aragona, 84131 Salerno, SA, Italy

**Keywords:** lower limb trauma, soft tissue defect, lower extremity reconstruction, split thickness skin graft, acellular dermal matrix, ADM, negative pressure wound therapy, NPWT, hyperbaric oxygen therapy, HBOT

## Abstract

**Background**: Distal lower extremity reconstruction is challenging. This study aims to propose a protocol for the treatment of traumatic soft tissue defects. The key concept is to combine the surgical armamentarium of the reconstructive surgeon with the advantages provided by hyperbaric oxygen therapy. **Methods**: This retrospective study analyzed data of 57 patients affected with unilateral or bilateral lower extremity trauma distal to the knee and involving soft tissues with no indication of immediate reconstruction between 2010 and 2021. Before the reconstructive procedure, all the patients underwent a stick swab procedure for the collection of microbiological samples and debridement. Patients were divided into two treatment groups and only one group underwent a combined therapeutic procedure with hyperbaric oxygen therapy. Negative pressure wound therapy (NPWT) was employed only if deemed necessary according to the defect’s depth and wound exudate. Surgical techniques, outcomes, and complications were discussed. **Results**: All patients achieved a complete recovery with no major complications and only minor complications observed. The study group treated with HBOT had a lower complication rate and lower percentages of minimal and partial graft loss compared with the same complications observed in the control group. No patients experienced HBOT-related complications. Significant reductions in the time to complete healing and the time from reconstruction to healing were found (*p* = 0.002 and *p* < 0.00001, respectively). **Conclusions**: A lower complication rate was observed in the group treated with HBOT. The administration of HBOT prior to soft tissue reconstruction significantly reduced the time to complete healing and the time interval from skin grafting to healing. However, prospective studies and randomized trials with larger cohorts should be designed to investigate the efficacy of HBOT for the treatment of lower extremity injuries with extensive soft tissue defects.

## 1. Introduction

Limb trauma is often associated with severe damage to the skin, subcutaneous tissue, and fascia, and may be accompanied by extensive damage to deep limb structures, such as muscles, vascular bundles, or bones, with possible functional and aesthetic sequelae; lymphedema is also a concerning condition following a trauma [1,2,3]. The treatment of major traumas of the lower limb has always been a major challenge for the reconstructive surgeon. Moreover, below the knee, any trauma could potentially lead to an exposure of deeper structures, i.e., tendons and bones. An additional drawback is the paucity of local soft tissue which may lead to the reconstructive management of these wounds being quite tough [4]. Many factors must be taken into account when designing a reconstructive plan for a patient with a soft tissue defect, including the anatomical defect, general condition of the patient, concomitant injuries, surgeon’s experience, patient occupation, and socioeconomic circumstances [5,6,7,8].

Soft tissue coverage, and therefore the reconstitution of the skin integrity, should be achieved as quickly as possible to minimize the risk of infection, avoid drying of the deep structures, and protect the neurovascular bundles [9]. The timing of soft tissue reconstruction after lower extremity trauma is not well detailed in the literature. It is defined as preferably within 2 weeks of the injury; however, the patient should be medically optimized and the wounds clean [1,10]. If this is not the case, it is advisable to delay the time to reconstruction by employing neoadjuvant therapies such as hyperbaric oxygen (HBO) therapy or vacuum-assisted closure (VAC) therapy, and to perform further debridement to clean the wound [10]. A clean wound that is free of necrotic tissue and contamination is of primary concern; rapid reconstruction of the soft tissues without regard to this tenet could inhibit healing [11,12].

The healing process of a wound depends on oxygen. In fact, all wounds that do not heal are hypoxic and more prone to infections. Hypoxia slows the synthesis and metabolism of collagen proteins; moreover, the migration and intervention of macrophages and consequently the formation of granulation tissue is delayed. The oxygen used in hyperbaric oxygen therapy (HBOT) enhances the healing of the soft tissue [13]. In patients with crush injuries, hyperoxygenation reduces edema, controls infection and collagen formation, and limits free radicals and reperfusion injury, thereby promoting healing [14].

Enhancing the rate of graft take is a challenging issue, especially in the sequelae of extensive soft tissue trauma. Contamination, difficulty in achieving a uniform bolster dressing for large defects, or patient compliance are tough concerns to deal with.

A critical review of our experience with complex soft tissue traumas of the lower extremity was performed, and our multidisciplinary reconstructive approach was detailed. To the best of our knowledge, this is the first defined reconstructive protocol that includes hyperbaric oxygen therapy as a neoadjuvant approach. The key concept is to combine the surgical reconstruction of soft tissues with the advantages provided by hyperbaric oxygen therapy.

## 2. Materials and Methods

The clinical records of extensive traumas of the lower limb undergoing delayed treatment at our university hospital were retrospectively reviewed from 2010 to 2021. A cohort of 57 patients was identified retrospectively and analyzed regarding the use of adjuvant HBO therapy (study group; *n* = 36) or the absence of adjuvant HBO therapy (control group; *n* = 21). All patients affected with unilateral or bilateral lower extremity soft tissue trauma distal to the knee were included. Patients were included in the study if they met the following criteria: acute soft tissue injury to the lower extremity distal to the knee, ability to understand the procedures, and ability to read and understand the informed consent information. Individuals who had undergone immediate reconstruction and those with a history of peripheral arterial disease or an exposed fracture were excluded. The medical records were acquired and analyzed retrospectively and epidemiological data and information regarding the trauma conditions, type and extent of the defect, the therapeutic approach, postoperative outcomes, the mean trauma-to-surgery and surgery-to-healing times, and complications were reviewed (Table 1 and Table 2).

After the first evaluation, all the patients underwent a stick swab procedure for the collection of microbiological samples and a surgical debridement. Negative pressure wound therapy (NPWT) was employed only if deemed necessary according to the defect’s depth and wound exudate. The placement of a split thickness skin graft (STSG) was the final reconstructive procedure in all cases. In the therapeutic management of this type of trauma, we gradually moved on to the combined use of HBOT. Consequently, the study group underwent a staged therapeutic protocol: after debridement, multiple cycles of hyperbaric oxygen therapy were offered before soft tissue reconstruction.

Follow-up was carried out for at least 12 months after the last surgical stage.

### Statistical Analysis

Statistical analyses were conducted using SPSS software (v25, IBM Corp., Armonk, NY, USA). The homogeneity between study groups was evaluated by the Z-test. The values for categorical variables were analyzed by the chi-squared test; Yates correction was applied. The values for quantitative variables were analyzed by the two-tail Mann–Whitney test. Significance was set at a value of *p* < 0.05.

## 3. Results

The study group consisted of 36 patients with a mean age of 38.4 ± 11.3 years. The etiology of traumatic events was a road accident in 21 cases (58%) and a work accident in 15 cases (42%). In 21 cases, the lower leg was involved, in eight cases, the foot was involved, and in seven cases, both regions were involved; in four cases, the trauma was bilateral. In all cases, there was extensive soft tissue involvement. At least one closed fracture was reported in nine patients (25%). The control group comprised 21 patients with a mean age of 31 ± 5.9 years.

All patients underwent debridement. The patients in the study group underwent multiple cycles of hyperbaric oxygen therapy, with an average 24.8 ± 5.5 sessions, during inpatient stay and after discharge. HBOT was administered on a daily basis according to relative requirements (one or two sessions per day). The choice of one or two sessions of HBOT was dictated by the initial conditions of the wounds. We discussed the methods for carrying out hyperbaric oxygen therapy with the anesthetist in charge of hyperbaric therapy, evaluating the options on a patient-by-patient basis. However, almost all patients started with two hyperbaric sessions a day and then moved on to just one hyperbaric session a day. Each single session provided oxygen at a pressure of 2.5 ATA for about 80–90 min. A second debridement session was performed if deemed necessary. NPWT was used in both the study and the control groups in 29 cases and 17 cases, respectively, with the application of negative pressure (−120 mmHg) for 10–12 days. The distribution of NPWT among the two groups was homogeneous (*p* = 0.97). In all cases, a split thickness skin graft was performed as the reconstructive procedure. In the study group, the reconstruction was performed in one surgical stage in 31 patients; five patients underwent a two-stage reconstruction with an acellular dermal matrix followed by a split thickness skin graft in a later surgical stage. In five cases, a meshed graft was necessary to expand the surface.

**Figure 1:**
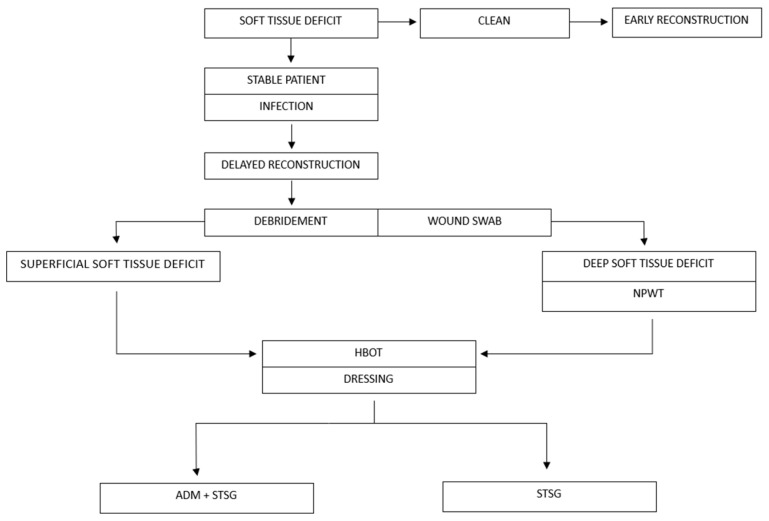
Authors’ protocol. NPWT—negative pressure wound therapy; HBOT—hyperbaric oxygen therapy; ADM—acellular dermal matrix; STSG—split thickness skin graft.

**Figure 2:**
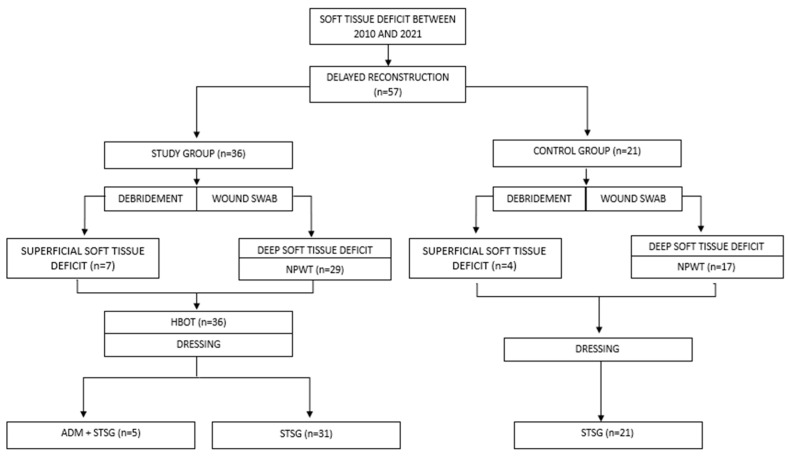
Study design. NPWT—negative pressure wound therapy; HBOT—hyperbaric oxygen therapy; ADM—acellular dermal matrix; STSG—split thickness skin graft.

In the operating room, all partial thickness grafts were dressed using moulage and a compressive dressing. All patients were then subjected to outpatient medication cycles for a variable number of sessions, with simple or advanced dressings as needed, to evaluate the outcomes until complete healing. The moulage was removed approximately seven days after the operation. In cases of reconstruction with the dermal matrix (only in the study group, *n* = 5), the graft was exposed and the silicone casing that surmounts the matrix was removed seven days after the operation. Subsequently, approximately two weeks after the first surgery, these patients underwent a second reconstructive operation. In all five cases, the second reconstructive operation consisted of an STSG placement. All patients underwent a course of antibiotic therapy that began in the operating room and continued for at least 7 days. The authors’ protocol is detailed in Figure 1 and the study design is depicted in Figure 2.

Major complications, namely sepsis, complete graft loss, and damage to the deep structures of the limb, were not reported.

Regarding the study group, in three cases (9%) minimal skin graft necrosis < 1 cm was observed; this was healed with dressings in an outpatient setting. In four cases (11%), a surgical revision was deemed necessary to speed up the healing process. Seven patients reported at least one complication; 69% of the patients did not report any complications. The most frequent complication was infection associated with partial graft necrosis; it was observed in four cases (11%) and in these cases, skin grafting was repeated after partial failure. No patients presented with HBOT-related complications (e.g., cerebral oxygen toxicity and barotrauma).

Conversely, the control group presented three cases of minimal graft losses (14%) and five cases (24%) of partial graft loss with recipient site infection (Table 2).

In the study group, the average trauma-to-surgery interval was 14.5 ± 3.1 days and between the surgery and healing was 19.2 ± 9 days. Overall, the time to complete healing was 33.1 ± 11.4 days. Conversely, in the control group, the average time elapsed from the trauma to the reconstructive procedure was 11.6 ± 1.9 days and the average time from the reconstructive surgery to complete healing was 28.8 ± 10 days. The time to complete healing was 39.9 ± 10.9 days. Significant reductions in the time to complete healing and the time from reconstruction to healing were found in the study group; the *p*-values are *p* = 0.002 and *p* < 0.00001, respectively. On the other hand, the time from the trauma to reconstruction was significantly prolonged in the study group (*p* = 0.0006).

Three-, six-, and twelve-month follow-ups were performed.

## 4. Discussion

The events leading to soft tissue loss in the lower limb can be varied and include traumatic, infectious, neoplastic, iatrogenic, vascular, or systemic events [15]. Lower limb trauma is a common major health problem that requires special attention because it can have a dangerous impact on an individual’s quality of life [16]. A degloving injury of the lower limb could be accompanied by extensive damage to the deep structures, and associated with severe tissue injury and a high risk of complications such as wound infection and later, phlegmon. The establishment of new treatment strategies, including hyperbaric oxygen therapy (HBO), negative pressure wound therapy (NPWT), and acellular dermal matrix (ADM), has improved the outcomes of limb reconstruction [17].

HBO increases dissolved plasmatic oxygen tension. Its beneficial effects, including reduction in edema, control of blood vessel and collagen formation, and reduction in free radicals and reperfusion injury, help in healing patients with crush injuries. It also enhances fibroblast function, angiogenesis, and alterations to local tissue circulation, promoting the formation of granulation tissue [14,18,19]. HBO therapy, applied according to the Amsterdam Protocol, has been shown to be effective in limiting the incidence of wound infection and the spread of refractory infections. The ischemic environment after trauma and cell death poses a risk for the development of anaerobic infection [20,21]; HBOT promotes aerobic metabolism by providing a high supply of oxygen and resistance to anaerobic bacteria [22]. Delaying the start of HBO therapy is also associated with a significantly higher infection rate and highly complex reconstructions. Early HBO therapy reduces treatment costs by decreasing surgical times, number of dressing changes, and the amount of dressing material [23]. HBO is generally considered safe and with few side effects. The most common problem is barotrauma of the middle ear. For some patients, HBO therapy is difficult due to claustrophobia, which can be reduced by having an attendant inside the chamber (multiplace) or beside it (monoplace). The only absolute contra-indications are an untreated pneumothorax and certain anticancer drugs because HBO significantly increases their cytotoxicity [23].

Moreover, most studies showed that early use of adjuvant HBO therapy contributes to graft and flap survival and provides a more favorable outcome [20,24,25]. On the other hand, the literature highlights that although there appears to be significant animal data to support the use of hyperbaric oxygen to prepare wounds for skin grafts, there are no clinical data to support this neoadjuvant approach. Perrins [26] conducted a blinded randomized study in which 24 patients received postoperative hyperbaric oxygen therapy and 24 patients were controls. Complete grafting occurred in 64% of patients treated with hyperbaric oxygen therapy and only 17% of controls, demonstrating the actual benefit of postoperative HBOT, but not its usefulness in the preoperative period. Furthermore, several published data sources address the use of HBO as adjuvant therapy in compromised flaps and grafts [11,23,26,27,28].

In summary, there are no important clinical data specifically demonstrating improved skin graft survival after preoperative use of hyperbaric oxygen, but in our clinical experience, HBO has given excellent results in this setting. The use of HBOT brought advantages in terms of healing of the graft, infection, and complication control, and favored less invasive and less complex reconstructions. In fact, several studies have demonstrated that the main cause of graft loss is infection [28,29,30]. Unal et al. [29], in a study of 132 patients, found that infection leads to graft loss in 23.7% of cases. Høgsberg et al. [31] reported a skin graft healing rate of only 33% in the presence of Pseudomonas aeruginosa compared with 77% in the absence of infection. Both studies have statistically significant results, but did not take hyperbaric therapy into consideration in their therapeutic process [29,31]. On the other hand, Roje et al. [24] found that HBO therapy, administered in an adjuvant setting, significantly reduced the frequency of deep soft tissue infections, osteomyelitis, skin graft loss, and flap necrosis in patients with complex Gustilo type III war wounds. In their study, deep soft tissue infection developed in 196 (68%) of 289 patients who did not receive adjuvant HBO therapy and in 35 (35%) of 99 patients who did receive adjuvant HBO therapy. The same study [20] also proved that HBO reduces the time to granulation formation and stimulates sufficient angiogenesis to support the take of a skin graft, which is a prerequisite for early surgical reconstruction, especially in patients with inadequately vascularized recipient sites. Roje and colleagues also proved that 151 (52% of 289) patients who did not receive HBO underwent skin graft loss and only 23 (23% of 99) who underwent HBO experienced the same issue. It should be noted that in their study, the clinical condition of the cohorts was different and HBOT was administered after surgical treatment.

Regarding postoperative complications, in our study, the HBOT group had even lower rates for both graft loss and infection; in fact, minimal graft loss and partial graft loss/infection were observed in 9% (*n* = 3) and 11% (*n* = 4) of patients, respectively. These rates are lower than those observed for the same complications in the control group (14% and 24%, respectively) and these data support the use of neoadjuvant HBOT, although our results are not statistically significant. Therefore, we could say that HBOT has been proven to be a protective factor against the occurrence of minimal and partial necrosis of the graft, even if its effect is not statistically significant.

Furthermore, significant reductions in time to complete healing and time from reconstruction to healing were found in the study group (both *p* < 0.05). This shows that HBOT could positively influence the reduction in healing times and related hospitalization and time to return to work; unfortunately, this latter variable was not evaluated. On the other hand, in the study group, the time from traumatic event to reconstruction was significantly prolonged (*p* = 0.0006); this finding may be due to the time dedicated to HBOT sessions. However, overall, time to complete healing was not affected; in our opinion, this may be due to the healing boost associated with oxygen therapy. Roje and colleagues did not evaluate the time elapsed between trauma and reconstruction or between reconstruction and healing, but only compared the median time to granulation formation between patients who received HBO therapy and those who did not receive it.

However, we must highlight one of the disadvantages of hyperbaric therapy: the cost. The cost of hyperbaric oxygen treatment at an average US hospital is USD 1800.00 for a 90 min HBOT session; also, not all hospitals have a hyperbaric chamber [14,32].

The introduction of NPWT has been proven to be an essential therapeutic advancement for the temporization of definitive soft tissue coverage and has increased the window within which acute reconstructions can be successfully performed. NPWT has extended the time to definitive soft tissue coverage beyond the dogmatic 72 h to weeks or even months and improves graft take by fluid removal, formation of granulation tissue, and reducing the risk of infection [33]. In our study, the distribution of NPWT among the two groups was homogeneous (*p* = 0.97).

Finally, it is necessary to mention the use of the acellular dermal matrix (ADM). Five patients underwent two-stage reconstruction with an acellular dermal matrix followed by a split thickness skin graft in a subsequent surgical stage. There are several publications on the use of ADM; however, only a few large studies on its use in lower extremity defects are available [17,34]. The first acellular dermal matrix was engineered more than 30 years ago and has previously been widely used in the management of burn wounds. It has since proven to be effective in the surgical treatment of post-traumatic soft tissue defects, especially in cases where reconstruction with surgical flaps is unavailable or undesirable, in combination with a split thickness skin graft [17,35,36]. The acellular dermal matrix can also be combined with negative pressure therapy (NPWT). ADM used in combination with NPWT has been successfully employed to generate a well-vascularized neo-dermis in complex wound beds, allowing for subsequent application of skin grafts [33]. Molnar et al. [37] have demonstrated how NPWT favors the matrix incorporation mechanism, thus promoting vascular growth and populating the matrix with host fibroblasts. They obtained a vascularization of the matrix in about one week compared with the standard two/four weeks. While the literature mentions the use of NPWT in combination with dermal matrices, there is a paucity of data regarding the use of the matrix in combination with HBOT [38,39,40,41]. Our study could therefore pave the way for a new trend in the management of these patients, using hyperbaric therapy to prepare the wound bed to accommodate the matrix. However, further data are needed in favor of the combined use of HBOT with the dermal matrix to highlight the advantages of this combination. However, it must be noted that dermal matrices present important negative and limiting aspects, such as cost and longer hospital stays, that must also be considered [17,33,42]. This study was certainly limited by the small patient population and this is probably the main cause of the lack of statistical significance of certain outcome analyses. Another important limitation of our study is the lack of a standard procedure for hyperbaric oxygen therapy (i.e., number of sessions per day per patient). The number of daily sessions was not standard for each patient and was dependent on the initial condition of the soft tissue wounds and agreement with the anesthetist in charge of the hyperbaric chamber. Furthermore, the use of only ADM in the study cohort could be a potential confounder and further limitation of our study. Prospective studies and randomized trials should be designed to further investigate the clinical role of HBOT as a neoadjuvant factor for faster healing of soft tissue defects of lower limbs.

## 5. Conclusions

Hyperbaric oxygen therapy is a relevant tool to achieve good surgical outcomes, lower infection rates, and better graft take rates in the setting of lower limb soft tissue trauma. The administration of HBOT prior to soft tissue reconstruction significantly reduced the time to complete healing and the time interval from skin grafting to healing. However, prospective studies and randomized trials with larger cohorts should be designed to investigate the efficacy of HBOT for the treatment of lower extremity injuries with extensive soft tissue defects.

## Figures and Tables

**Table 1:** Patients and procedures.

Variable	HBOT Group Value (Rate)	Control Group Value (Rate)
Patients	36	21
Age, years Mean ± SD	38.4 ± 11.3 years	31 ± 5.9 years
Gender Female Male	6 (17%) 30 (83%)	5 (24%) 16 (76%)
Event Road accident Work accident	21 (58%) 15 (42%)	13 (38%) 8 (62%)
Trauma site Only the right leg Only the left leg The left leg and foot Both legs Right leg and foot The right leg and foot plus the left leg The left leg and foot plus the right leg Both feet	16 (44%) 11 (30%) 3 (8%) 2 (6%) 1 (3%) 1 (3%) 1 (3%) 1 (3%)	6 (29%) 6 (29%) 3 (14%) 2 (9%) 3 (14%) - - 1 (5%)
HBO sessions, (days)	24.8 ± 5.5	-
Reconstructive treatment STSG ADM + STSG	31 (86%) 5 (14%)	21 (100%) -
Follow-up (months)	3-6-9-12	3-6-9-12

Legend. HBO—hyperbaric oxygen therapy; ADM—acellular dermal matrix; STSG—split thickness skin graft.

**Table 2:** Outcomes evaluation.

Complications	Study Group			Control Group	*p*-Value
	STSG *N* (%)	STSG MESHED *N* (%)	ADM + STSG *N* (%)	STSG *N* (%)	
Minimal graft loss Partial graft loss	2 (6%) 2 (6%)	- 1 (3%)	1 (3%) 1 (3%)	3 (14%) 5 (24%)	0.79 0.37
Infection Donor site Recipient site	6 (17%) 3 3	1 (3%) - 1	- - -	7 (33%) 2 (9%) 5 (24%)	
Surgical revision Skin graft	4 (11%)	-	-	5 (24%)	
Time (days)					
Time from event to reconstruction Time from reconstruction to healing Time to complete healing		14.5 ± 3.1 19.2 ± 9 33.1 ± 11.4		11.6 ± 1.9 28.8 ± 10 39.9 ± 10.9	0.0006 <0.00001 0.002

Legend. ADM—acellular dermal matrix; STSG—split thickness skin graft.

## Data Availability

The data presented in this study are available on request from the corresponding author.
